# Molecular Characterization of Enterotoxin-Producing *Escherichia coli* Collected in 2011–2012, Russia

**DOI:** 10.1371/journal.pone.0123357

**Published:** 2015-04-29

**Authors:** Nikolay N. Kartsev, Nadezhda K. Fursova, Dmitry M. Pachkunov, Vasiliy A. Bannov, Boris V. Eruslanov, Edward A. Svetoch, Ivan A. Dyatlov

**Affiliations:** 1 Department of Molecular Microbiology, State Research Center for Applied Microbiology and Biotechnology, Obolensk, Russian Federation; 2 Department of Innovation Research, Volga State Technological University, Yoshkar-Ola, Russian Federation; Ross University School of Veterinary Medicine, SAINT KITTS AND NEVIS

## Abstract

Enterotoxin-producing *Escherichia coli* (ETEC) are one of the main causative agents of diarrhea in children especially in developing countries and travel diarrhoea in adults. Pathogenic properties of ETEC associated with their ability to produce a heat-stable (ST) and/or heat-labile (LT) enterotoxins, as well as adhesins providing bacterial adhesion to intestinal epithelial cells. This study presents the molecular characterization of the ETEC isolates collected from the Central and Far-Eastern regions of Russia in 2011–2012. It was shown that all ETEC under study (n=18) had the heat-labile enterotoxin-coding operon *elt*, and had no the genes of the heat-stable enterotoxin operon *est*. DNA sequencing revealed two types of nucleotide exchanges in the *eltB* gene coding subunit B of LT in isolates collected from Cherepovets city (Central region, Russia) and Vladivostok city (Far-East region, Russia). Only one ETEC strain carried genes *cfaA*, *cfaB*, *cfaC* and *cfaD* coding adhesion factor CFA/I. Expression of LT in four ETEC isolates in the agglutination reaction was detected using a latex test-system. The isolates were assigned to serogroups O142 (n = 6), О6 (n = 4), О25 (n = 5), О26 (n = 2), and O115 (n = 1). Genotyping showed that they belonged to an earlier described sequence-type ST4 (n = 3) as well as to 11 novel sequence-types ST1043, ST1312, ST3697, ST3707, ST3708, ST3709, ST3710, ST3755, ST3756, ST3757 and ST4509. The ETEC isolates displayed different levels of antimicrobial resistance. Eight isolates were resistant to only one drug, three isolates—to two drugs, one isolate—to three drugs, two isolates—to four antibacterials, and only one isolate to each of the five, six and ten antibacterials simultaneously. Genetic determinants of the resistance to beta-lactams and other classes of antibacterials on the ETEC genomes were identified. There are *bla*_TEM_ (n = 10), *bla*_CTX-M-15_ (n = 1), class 1 integron (n = 3) carrying resistance cassettes to aminoglycosides and sulphonamides *dfrA17-aadA5* and *dfrA12-orfF-aadA2*. One isolate ETEC_Ef-6 was found to be a multidrug-resistant (MDR) pathogen that carried both the beta-lactamase gene and class 1 integron. These data suggest the circulation of ETEC in Russia. Further investigations are necessary to study the spread of the revealed ETEC sequence types (STs) and serotypes. Their role in the etiology of diarrhea should be also estimated.

## Introduction

*Escherichia coli* bacteria causing human intestinal infections are subdivided into six pathotypes, namely enterotoxigenic (ETEC), enteropathogenic (EPEC), enteroinvasive (EIEC), enterohaemorrhagic (EHEC), enteroaggregative (EAgEC) and diffusely-aggregative (DAEC) [1].

ETEC are the enteric pathogens that may cause cholera-like diarrhea in animals and humans. Of all pathogenic *E*. *coli* bacteria ETEC are the most common causative agents of human diarrhea worldwide. More than 650 million cases of ETEC-infections are registered annually in 2000s among which 800 thousands cases are fatal [2]. It is estimated that each year on the period from 2009 to 2012 nearly 600,000 children less than five years of age die from severe dehydrating diarrhea, mostly in the developing world [3]. Among the many causes of diarrheal disease, enterotoxigenic *E*. *coli* and *Shigella* are the two most important bacterial pathogens [4]. ETEC can also cause so called traveler’s diarrhea, the most frequent illness reported and related to people who traveled from an industrialized country to a developing country, including intestinal infections in the military contingent of the United Nations. The incidence of diarrhea among visitors of the tropics and subtropics varies from 10% to 60%. A high percentage of incidences are registered in Latin America, Africa and the Indian subcontinent [5]. The seasonal prevalence of ETEC-associated diarrhea has been established. The morbidity rate increases by 7% per each degree of rise in ambient temperature that is accompanied by the growth and spread of bacteria contaminating food and water [6].

The main molecular mechanisms of the ETEC pathogenicity imply the production of heat-stable (ST) and heat-labile (LT) enterotoxins disturbing the electrolyte balance in intestinal epithelial cells of the infected macroorganism, thereby inducing acute profuse diarrhea. The mechanisms rely also on the availability of colonization factors (CFs), providing attachment of bacteria to the intestinal epithelial cells. The ST belongs to membrane-damaging toxins encoded by the transposon-associated *estA* and *estB* genes localized on the plasmids. The LT is multimeric protein of 85.5 kDa similar to cholera toxin that consists of a single L_A_ enzyme-subunit (28.0 kDa) and a binding pentamer of L_B_ subunits (11.5 kDa). The LT activates adenylate cyclase and leads to an increase in levels of intracellular cyclic AMP (cAMP) following by stimulation of chloride secretion resulting diarrhea. The ST by binding to guanilate cyclase C stimulates increasing of intracellular cyclic guanosine monophosphate (cGMP) levels and chloride secretion resulting dehydrating diarrhea [7]. It is interesting that contribution of ST and LT enterotoxins to ETEC-infection etiology was changed for the last decades. If before the major ETEC outbreaks were mainly caused by ST-producing *E*. *coli*, the proportion of ST- and LT-producing strains became equal (30–35%) in 2000s [8], and quite recently the prevalence of LT-producing ETEC strains throughout the world has been reported. A ratio of ST- and LT-producing ETEC varies depending on a region. So, LT-producing strains prevail in traveling communities (38%) in Latin America and the Caribbean in contrast to non-traveling ones (30%) in Asian-Pacific countries [9]. In Russia LT-producing *E*. *coli* strains were detected in 36.0% of fecal samples collected from patients with acute and chronic intestinal diseases [10]. Over 90% ST-LT-producing strains isolated worldwide in 1980s had adhesins and colonization factors (CFs). More than 20 CFs with unique fimbrial primary structure have been described for today. CFA/I, CFA/II and CFA/IV are the most characterized ETEC colonization factors, composed from CS1—CS6 surface antigens [7]. Despite the important role of CFs in ETEC pathogenesis, currently these structures are being identified only in 50–70% isolates collected in the world [8]. It was shown that large part of phenotypically undetectable CFAs in LT-producing ETEC can be explained by the presence of undetectable CFAs variants with unknown structure [11]. It was found that ETEC colonization factor polycistronic operon coding fimbriae subunits, chaperones and Usher proteins can locate both on chromosome and plasmid [12].

ETEC strains isolated from humans belong to different O-groups, but more often (60–70% strains collected worldwide)—to O6, O8, O25, O78, O128, and O153 groups. The remaining 30–40% constitutes a great number of other serogroups [13]. ETEC isolates of O148 serogroup are also isolated in Russia [14]. PCR-serotyping of *E*. *coli* identifies the genes coding enzymes involved into the O-antigen synthesis. These determinants locate in the O-cluster formerly known as *rfb*-cluster coding *E*. *coli* housekeeping genes [15]. Genetic structures of microbial populations and a contribution of the specific clone to the development of the epidemiological situation are studied by various genotyping methods including ribotyping, Pulsed Field Gel Electrophoresis (PFGE), Random Amplified Polymorphic DNA (RAPD), and Multilocus Sequence Typing (MLST). The MLST analysis of bacterial isolates collected during an outbreak allows one to follow up their relationships (local epidemiology) and to establish their potential links with similar isolates from other geographic regions (global epidemiology). MLST algorithm developed for pathogenic *E*. *coli* is based on the polymorphism of seven chromosomal housekeeping genes: *adk* (adenylate kinase), *fumC* (fumarate hydratase), *gyrB* (DNA gyrase), *icd* (isocitrate/isopropylmalate dehydrogenase), *mdh* (malate dehydrogenase), *purA* (adenylosuccinate dehydrogenase), *recA* (ATP/GTP binding motif) (Mark Achtman Database, http://mlst.warwick.ac.uk/mlst/dbs/Ecoli). The genetic diversity of ETEC strains was explained by the ability of *E*. *coli* to capture virulence plasmids at rapid evolutionary processes and great structural variety of their chromosome [16]. The analysis of many strains from various sources collected worldwide confirmed the intensive exchange of enterotoxin and adhesin genes among ETEC of different genetic lineages [17].

The objective of this study is to characterize ETEC strains isolated during the food-borne outbreak in Cherepovets in August, 2011, as well as those collected during a sporadic case of food-borne infection in Vladivostok in September, 2012. ETEC phenotypes responsible for antimicrobial susceptibility and LT-production were determined. Genetic determinants coding enterotoxins and adhesins, and antibacterial resistance genes in their genomes were detected by PCR. O-groups and sequence types (ST) of the strains were identified.

## Material and Methods

### Ethics Statement

Our laboratory is a reference center for the study of food-borne infections agents, and we do not have direct contact with patients. Materials for research were obtained from the regional centers of Hygiene and Epidemiology. The study was not reviewed and approved by an institutional review board (ethics committee) before the study began because we studied *E*. *coli* strains, isolated from the fecal samples without specifying the names of patients, the race/ethnicity, age, religion, sex/gender, sexual orientation, or other socially constructed groupings. Our task was to identify the agent of food-borne outbreak and identify the source in food. However, in accordance with the rules of the Russian Federation, every patient entering the hospital signed informed consent to medical procedures and diagnostic tests.

### Microbiology

#### Bacterial isolates and strains

LT-positive ETEC isolates were collected from the sick people with moderate gastroenteritis during an outbreak of food-borne infection in Cherepovets (Central Region of Russia) in August, 2011 (n = 12) and from a patient with moderate food-borne infection in Vladivostok (Far Eastern region of Russia) in September, 2012 (n = 6).

#### Bacterial isolation and growing

Pathogenic *E*. *coli* were isolated from the fecal and food samples using nutrient enrichment mediums «Nutrient medium No. 11 GRM», «RVS-broth», «Nutrient medium No. GRM», «SDS-broth» (SRCAMB, Obolensk, Russia) followed by growing on the selective agar mediums «Nutrient medium No. 1 GRM», «*E*. *coli* O157:H7 Sorbitol-agar », «XLD-agar», «Iron-glucose-lactose agar with urea», «Endo-agar GRM» (SRCAMB, Obolensk, Russia). Identified *E*. *coli* strains were grown on agar nutrient media «Muller-Hinton» (Himedia, India), «Luria Bertani broth» (Difco, USA), and «Nutrient medium No. 1 GRM» (SRCAMB, Obolensk, Russia). Bacterial isolates were stored in 10% glycerol at minus 70 degrees on Celsius.

#### LT induction

Mundell nutrient medium [18] was inoculated by 10^9^ CFU/ml *E*. *coli* bacterial suspension in proportion 10:1, than incubated at aeration (120 rpm/min) at 37 degrees on Celsius for 24 h. After that 25 μl of polymixin B (10000 U/ml) was added into 1 ml bacterial culture and incubated without aeration at 37 degrees on Celsius for 4 h. Bacterial cells were removed by centrifugation at 900 g for 20 min. Super was filtrated using «Ultrafree-MC Microcentrifuge Filters» (Sigma-Aldrich, CIIIA) with pores 0,22 μm. Obtained filtrate was the object for LT detection using latex-agglutination assay (SRCAMB, Obolensk, Russia).

#### Latex agglutination

Latex agglutination was done by mixing 20 μl of the bacterial filtrate and 20 μl of the latex suspension for LT detection. Pure cholera toxin (Oxoid, UK) in concentration of 2 mg/ml was used as positive control. Bacterial filtrate of the non-LT-producing *E*. *coli* strain HB101 was used as negative control.

#### Susceptibility to antibacterials

Susceptibility to antibacterials (ABs) was measured by disk-diffusion method and microdilution method in broth according to EUCAST guidelines (http://www.eucast.org/clinical_breakpoints/). *E*. *coli* strain ATCC 25922 was used as control.

### Molecular Genetics Methods

#### Detection of pathogenic *E*. *coli* in clinical samples by real-time PCR

Preliminary detection of ETEC, EPEC, EHEC, EAgEC and EIEC in clinical specimens, samples of food and enrichment cultures of pathogenic of *E*. *coli* was done using commercial assay “AmpliSens Escherichioses-FRT” (InterLabService, Russia). The assay is based on the amplification of pathogen genome specific region using specific *E*. *coli* primers and oligonucleotide probes. The amplified product is detected in real-time PCR (RT-PCR). The kit is a qualitative test that contains the internal control (http://www.interlabservice.ru/en/catalog/index.php?sid=1104&id=8415).

#### Detection of the resistance genetic determinants

Beta-lactamase TEM- and CTX-M-type genes as well as class 1 and 2 integron were detected by PCR with specific primers [19].

#### Bacterial strain serotyping

ETEC serotyping was done both by i) agglutination with serums of the kits “OK-polyvalent Escherichia serums for agglutination» and «O-serogroup Escherichia serums for agglutination” (Biomed, Russia), and ii) PCR-serotyping using specific oligonucleotide primers [20–22] in 25 μl reaction mix consisting of 2.5 μl 10×*Taq*-buffer with (NH_4_)_2_SO_4_ and 20 mM MgCl_2_, 2.5 μl of 2.5 mM solution of each dNTPs, 0.25 μl of BSA, 12.5 pM each primer, 0.7 U of recombinant *Taq*-polymerase, and 5 μl of the bacterial lysate at the program consisting of initial denaturation for 5 min at 95°C; then 30 cycles of denaturation for 30 sec at 95°C, annealing for 30 sec at 58°C, and elongation for 40 sec at 72°C; final elongation for 5 min at 72°C.

#### Virulence genes detection

ST- and LT-coding genes as well as CFA/I-coding genes were detected by PCR. Design of the specific primers was done by Vector NTI Advance 11.5 software (Invitrogen, USA). DNA amplification was carried in 25 μl of the reaction mixture contained of 2.5 μl of the 10×*Taq*-buffer (NH_4_)_2_SO_4_ and 20 mM MgCl_2_, 2.5 μl of the 2,5 mM solutions of each dNTPs, 0.5 μl BSA, 12.5 pM each primer, 0.7 U recombinant *Taq*-polymerase and 5 μl cell lysate at the program including initial denaturation for 5 min at 95°C; then 30 cycles of denaturation at 45 sec for 95°C, annealing for 45 sec at appropriate Ta°C (Table 1) and elongation for 45 sec at 72°C; final elongation for 5 min at 72°C.

**Table 1:** Oligonucleotide primers for enterotoxin and adhesin genes detection.

Target gene (PCR-product size, bp)	Primer	Oligonucleotide sequence 5’-3’	Ta, °C
*eltA*—Heat-labile enterotoxin subunit A gene (362)	**ltA-F**	TGCACATGGCGACAAATTATACCG	55
	**ltA-R**	GTACTCCACCTAACGCAGAAACC	55
*eltB*—Heat-labile enterotoxin subunit B gene (271)	**ltB-F**	GGGTTATTTACGGCGTTACTATCC	54
	**ltB1-R**	GGGGTGTGAATCTTAATGTGTCC	54
*eltAB*—Heat-labile enterotoxin operon (995^a^)	**ltA-F**	TGCACATGGCGACAAATTATACCG	58
	**ltB3-R**	TCCTTCCTCCTTTCAATGGC	58
*st*—Heat-stable enterotoxin gene (380)	**STa2-F**	CGTCGTGTTTCGGAGGTAATATGA	55
	**STa2-R**	TGCCTTCCGCTAACACTTCC	55
*cfaA*—Fimbrial subunit A gene (464)	**cfaA-F**	AGAAAACAATAGGCGCAATGG	54
	**cfaA2-R**	TTGACCAGCTGTTAGTGCGC	54
*cfaB*—Fimbrial subunit B (periplasmic chaperone) gene (637)	**cfaB-F**	CGACGGCGAACTTTATGATCTATCC	59
	**cfaB2-R**	CCCGCGCTTCCATCAACACT	59
*cfaC*—Fimbrial subunit C gene (1163)	**cfaC1-F**	ACCACCGTGGCATTTGAGTCTCC	60
	**cfaC1-R**	GGGAAGCAGAAACCATCAGTATAGG	60
*cfaD*—Fimbrial subunit D (positive expression regulator) gene (929)	**cfaD-F**	TATTTTGTGCGGTGGTCAGTGC	59
	**cfaD-R**	GCCTAGCATCTGCGGTTACTCG	59

Note:

^a^ product size varies depending on *eltB* gene variability

#### Plasmid DNA extraction

DNA extraction was done by alkaline method [23]. Plasmid profiles were analyzed by electrophoresis at 0.8% agarose gel.

#### Strains genotyping

Strains genotyping was done by RAPD-PCR using «random» primer OPA11 [24]. DNA amplification was carried in 25 μl of the reaction mixture contained 2.5 μl of 10×*Taq*-buffer with (NH_4_)_2_SO_4_ and 20 mM MgCl_2_, 2.5 μl of 2.5 mM solution of each dNTPs, 20 pM of each primer, 0.7 U of the recombinant *Taq*-polymerase, and 5 μl of cell lysate at the program involving initial denaturation for 3 min at 95°C; 5 cycles of denaturation for 1 min at 94°C, annealing for 1 min at 35°C, and elongation for 1 min at 72°C; then 40 cycles including denaturation for 30 sec at 94°C, annealing for 30 sec at 35°C, and elongation for 2 min at 72°C, then final elongation for 10 min at 72°C.

#### Multi Locus Sequence Typing

MLST of the ETEC strains was done using sequencing of seven house-keeping genes, namely *adk* (adenylate kinase), *fumC* (fumarate hydratase), *gyrB* (DNA gyrase), *icd* (isocitrate/isopropylmalate dehydrogenase), *mdh* (malate dehydrogenase), *purA* (adenyl-succinate dehydrogenase), and *recA* (ATP/GTP-binding motif) according the approach presented by web-site of the MLST.UCC Mark Achtman database (http://mlst.warwick.ac.uk/mlst/dbs/Ecoli/). DNA sequence analysis was done by Vector NTI Advance 11.5 (Invitrogen, USA), BLAST (http://blast.ncbi.nlm.nih.gov/Blast.cgi?PROGRAM=blastn&PAGE_TYPE=BlastSearch&LINK_LOC=blasthome), and Chromas Version 1.5 (Technelysium Ply Ltd, Australia).

#### Phylogenetic analysis

Dendrogram showing the genetic relationship of the ETEC strains under study was constructed based on MLST sequences by Mega6 software. Phylogenetic reconstruction was performed using «neighbor-joining» method based on a two-parameter model nucleotide changes, branches were generated by bootstrapping with 1000 replications [25].

## Results and Discussion

Heat-labile enterotoxin-producing *E*. *coli* strains collected during an outbreak of food-borne infection in Cherepovets (Central Region, Russia) in August, 2011 (n = 12) and from a patient with moderate food-borne infection in Vladivostok (Far Eastern region, Russia) in September, 2012 (n = 6) are characterized in this study.

The outbreak in Cherepovets involved workers who had taken their meal from the factory canteen. Mild disease developed in 97 infected persons, while moderate disease (abdominal pain, multiple liquid stools, vomiting, and temperature of 37°C to 38°C) was diagnosed in 10 patients. Totally, 76 clinical samples comprising 31 fecal samples from sick persons, 19 fecal samples from healthy canteen personnel, and 26 samples of foodstuffs (vegetables, greens and dairy products) have been analyzed. The preliminary analysis of the clinical materials and foodstuffs by the RT-PCR revealed the presence of DNAs of pathogenic *E*. *coli* bacteria, namely ETEC (n = 23), EPEC (n = 18), EAgEC (n = 5), and EIEC (n = 1) in the samples. Totally 70 *E*. *coli* isolates were collected. All of them were tested on belonging to the listed above *E*. *coli* pathogenic groups including presence of heat labile toxin *elt* and heat stable toxin *est* genes. As a result 11 ETEC strains were isolated from nine patients’ clinical material, and one ETEC strain was isolated from a sample of sour cream (Table 2).

**Table 2:** Characteristics of ETEC isolates collected in 2011–2012.

ETEC Isolate	City	Patient	Isolation source	Accession in “SCPM-Obolensk”	O-group	*adk*	*fumC*	*icd*	*purA*	*gyrB*	*recA*	*mdh*	ST	Clonal complex	Antibacterial resistance	Resistance genes
**16-8V**	Ch	16	feces	B-7205	O25	66	11	223	8	4	2	8	3755	-	AMI CML	-
**22-2V**	Ch	22	feces	B-7203	O6	6	5	8	8	4	2	8	4	10	CML DOC	-
**24-10V**	Ch	24	feces	B-7204	O25	6	11	8	78	4	2	8	1312	-	CML	-
**26-15V**	Ch	26	feces	B-7196	O6	127	24	299	8	4	2	8	3756	-	AMP	*bla*_TEM_
**27-1V**	Ch	27	feces	B-7207	O25	66	11	8	78	4	2	298	3697	-	DOC	-
**73-7V**	Ch	73	feces	B-7199	O25	232	459	39	27	4	2	8	3708	-	AMP NAL TRM	-
**73-10V**	Ch	73	feces	B-7206	O26	56	11	299	78	4	2	8	3709	-	-	-
**85-4V**	Ch	85	feces	B7202	O26	332	11	299	8	331	2	8	3757	-	AMI AMP CML DOC NAL TRM	*bla*_TEM_
**85-17V**	Ch	85	feces	B-7197	O115^a^	232	37	25	5	29	73	4	4509	-	AMI CML	*int1-dfrA17-aadA5*
**112-2V**	Ch	-	sour cream	B-7201	O6	6	5	8	8	4	2	8	4	10	AMI AMP DOC NAL	*bla*_TEM_
**118-5V**	Ch	118	feces	B-7198	O6	232	68	223	8	347	6	8	3710	-	AMP	*bla*_TEM_
**121-3V**	Ch	121	feces	B-7200	O25	10	11	8	78	4	2	8	1043	-	AMP CML CTX DOC	*bla*_TEM_
**Ef-2**	V	Ef	feces	B-7684	O142	ND	ND	ND	ND	ND	ND	ND	ND	ND	AMP	*bla*_TEM_
**Ef-4**	V	Ef	feces	B-7685	O142	ND	ND	ND	ND	ND	ND	ND	ND	ND	AMP	*bla*_TEM_
**Ef-5**	V	Ef	feces	B-7686	O142	ND	ND	ND	ND	ND	ND	ND	ND	ND	AMP	*bla*_TEM_
**Ef-6**	V	Ef	feces	B-7687	O142	6	19	16	5	15	44	9	3707	-	AMI AMP CAZ CIP CTA CTX DOC FEP GEN TRM	*bla*_TEM_, *bla*_CTX-M-15_ *int1-dfrA12-orfF-aadA2*
**Ef-7**	V	Ef	feces	B-7688	O142	6	5	8	8	4	2	8	4	10	AMI AMP DOC GEN TRM	*bla*_TEM_ *int1*
**Ef-9**	V	Ef	feces	B-7689	O142	ND	ND	ND	ND	ND	ND	ND	ND	ND	AMP	*bla*_TEM_

Note: Ch—Cherepovets; V—Vladivostok; “SCPM-Obolensk”—The Strain Collection of Pathogenic Microorganisms of the State Research Center for Applied Microbiology and Biotechnology; AMI—amikacin; AMP—ampicillin; CAZ—ceftazidime; CIP—ciprofloxacin; CML—chloramphenicol; CTA—ceftriaxone; CTX—cefotaxime; DOC—doxycycline; NAL—nalidixic acid; TRM—trimethoprim; FEP—cefepime; GEN—gentamicin; ND—not detected; «-»—no

^a^ data obtained by serotyping without confirmation by PCR

A sporadic case of the moderate food-borne infection was registered in Vladivostok in September, 2012. A patient complained of an abdominal pain, diarrhea, vomiting, and fever. ETEC DNA was identified in clinical specimens (feces) by means of the RT-PCR test system "AmpliSens Escherihioses-FRT». The presence of heat labile enterotoxin genes *eltA* and *eltB* in 6 of 10 *E*. *coli* isolates (ETEC_Ef-2, ETEC_Ef-4, ETEC_Ef-5, ETEC_Ef-6, ETEC_Ef-7 and ETEC_Ef-9) was confirmed by the conventional PCR (Table 2).

To determine genetic structure of main ETEC pathogenicity factors, a set of specific primers including those to detect A and B subunits of heat-labile enterotoxin genes *eltA* and *eltB*, complete operon *elt*, as well as A subunit of heat-stable enterotoxin gene *estA*, and subunits of the adhesion factor CFA/I (*cfaA*, *cfaB*, *cfaC* and *cfaD*) were designed (Table 1).

**Fig 1:**
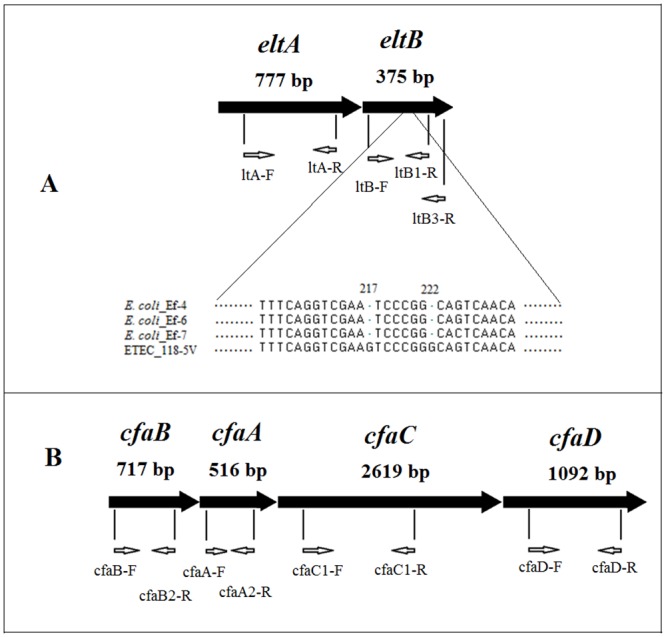
Primer arrangement on the genes. **A**—the *eltA* and the *eltB* genes; alignment of the *eltB* gene nucleotides from four *E*. *coli* isolates. **B**—CFA/I operon consisting of the *cfaA*, *cfaB*, *cfaC* and *cfaC* genes. Nucleotide numbers indicate their position from the start of the open reading frame.

It was shown that all ETEC isolates collected from the food-borne outbreak and the sporadic case in Cherepovets and Vladivostok respectively, had the complete length copy of the *elt* operon (Fig 1), and did not carry the heat-stable enterotoxin *estA* gene. Sequence of PCR products of *eltA* and *eltB* genes amplified using primers LT-A(For) and LT-B3(Rev) has shown differences between nucleotide sequences of the *elt* operon in the strain ETEC_118-5V (GenBank: JX504011) (clinical specimens; Cherepovets, 2011) and those of the *elt* operon in the ETEC_Ef-4 (GenBank: KF733766), ETEC_Ef-6 (GenBank: KF733765) and ETEC_Ef-7 (GenBank: KF733767) strains (Vladivostok, 2012). In the subunit B gene there were found differences in nucleotide positions 217 and 222 (Fig 1), reflecting the known fact of this gene heterogeneity.

From one to six molecular weight-varying plasmids were found in ETEC strains under study. However, the *elt* operon in these strains is likely to locate not on a plasmid as described in some studies [2], but in a chromosome because it was not transmitted via conjugation. This observation agrees with data from retrospective molecular genetic analyses of *elt* operons of ETEC strains collected from numerous sources worldwide. It has been shown that the *elt* locus may locate inside Lambda prophages [26].

Heat-labile enterotoxin gene expression in ETEC_27–1, ETEC_73–7, ETEC_Ef-4 and ETEC_Ef-6 strains was confirmed in latex agglutination tests. The LT production revealed in the strains was some lower compared to the control (purified cholera toxin, 2 g/L). The LT was not identified in other ETEC strains that indicate a low level of expression.

Genetic structures of the *cfa* operon, whose products are responsible for ETEC adhesion in humans (*cfaA*, *cfaB*, *cfaC* and *cfaD*) (Fig 1), were detected in the only strain ETEC_118-5V isolated from the patient in Cherepovets in 2011. Nucleotide sequences of *cfaA*, *cfaB*, *cfaC*, and *cfaD* genes were submitted to the GenBank database (JX504012, JX504013, JX504014, and JX504015, correspondingly). The discovery of the adhesion genes in only one ETEC isolate can be explained by the genetic heterogeneity of adhesion and colonization determinants. It is also possible that the only set of specific PCR primers designed for a specific CFA-type fails to detect these patterns.

Isolates obtained in Cherepovets were attributed to О6 (n = 3), О25 (n = 5) and О26 (n = 2) groups by serotyping using poly- and monovalent agglutinating sera as well as by serotyping using PCR with specific primers for the flippase *wzx* gene and the O-antigen polymerase *wzy* gene. Isolates obtained in Vladivostok were attributed to О142 group (Table 2). *E*. *coli* of O6, O25, O115 and O142 groups identified in the research are typical representatives of ETEC strains being also isolated in some other countries, e.g. in China, Japan, and Bangladesh [27–29]. Before the research there were reported about identification of ETEC of О148 serogroup in Russia [14]. Of special interest is identification of the ETEC strains belonging to O26 group that is more common for Shiga toxin-producing *E*. *coli* (STEC). Such ETEC strains were isolated not so often in the world, one example is isolation of O26 ETEC from bulls Mithun in India were reported in 2009 [30].

**Fig 2:**
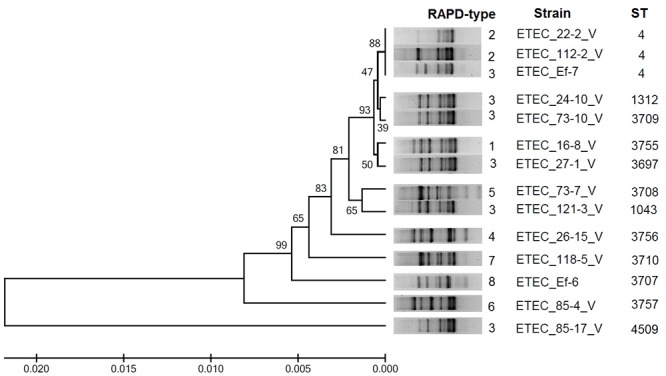
MLST phylogenetic tree of the 14 ETEC strains as reconstructed from the sequences of seven “housekeeping” genes. Phylogenetic tree constructed after assembly and alignment of MLST DNA sequences using the Mega 6 program. MLST and RAPD experiments are described in Materials and Method.

The MLST analysis of ETEC isolates under study revealed 12 different sequence-types. It was shown that two strains from Cherepovets (ETEC_22-2V and ETEC_112-2V) and one strain from Vladivostok (ETEC_Ef 7) belonged to the previously known sequence-type ST4 that had been presented in the MLST database by the ETEC strain (ETEC 117/86) isolated in Germany in 1986. It should be noted that the latter belongs to О6 serogroup as do our strains ETEC_22-2V and ETEC_112-2V isolated from two patients and sour cream sample during the outbreak in Cherepovets in 2011. It is noteworthy that the Clonal complex ST10 which ST4 sequence-type belongs to is quite typical for ETEC isolates worldwide [7]. The fact that ETEC strains of the sequence-type ST4 were isolated not only from human (ETEC_22-2V) but also from sour cream (ETEC_112-2V) allows one to consider sour cream as a potential source of the outbreak in Cherepovets. This observation agrees with earlier reports stating milk and dairy products as a potential reservoir for ETEC-associated infections. In addition to the sequence-type ST4, 11 novel sequence-types (ST1043, ST1312, ST3697, ST3707, ST3708, ST3709, ST3710, ST3755, ST3756, ST3757, and ST4509) were described in our research. They differ from each other and from the ST4 by sets of "housekeeping gene" alleles. The most remote from the ST4 is ST4509 of the strain ETEC_85-17V. It differs by all seven gene alleles. Other STs differ from the ST4 by two to six gene alleles. The *adk*, *fumC*, *icd* and *purA* genes were the most variable, while the *gyrB*, *recA* and *mdh* genes were more conservative (Table 2). It is interesting that some isolates that differed in their sequence-types, serogroups and resistance genes were collected from one and the same patient, for example, ETEC_85-4V and ETEC_85-17V strains, and ETEC_73-7V and ETEC_73-10V strains were isolated from the patient 85 and patient 73 respectively. The analysis of the phylogenic tree structure and RAPD data of the ETEC strains described in our study allow one to combine the ST4, ST1312, ST3709, ST3755, and ST3697 that are genetically related into one cluster, the ST3708 and ST1043 into other cluster, while remaining STs were unclustered (Fig 2).

It was shown that the ETEC strains had very different spectra of antibacterial susceptibility to antibiotics of several functional classes. Eight isolates were resistant to one drug, three isolates—to two drugs, one isolate—to three drugs, two isolates—to four drugs, one isolate—to five drugs, one isolate—to six drugs, and one isolate—to ten drugs (Table 2). The genetic determinants of the beta-lactam resistance were detected in six ETEC strains. Namely they were beta-lactamase of TEM-type gene (ETEC_26-15V, ETEC_85-4V, ETEC_112-2V, ETEC_118-5V, ETEC_121-3V, and ETEC_Ef-6 strains) and the epidemiologically significant *bla*_CTX-M-15_ gene (ETEC_Ef-6 strain). Thus, it is shown that the *bla*_CTX-M-15_ gene is present in *E*. *coli* bacteria of the novel ST3707 sequence-type. It should be noted that CTX-M is the main type of extended spectrum beta-lactamases (ESBLs) in *Enterobacteriaceae*, while they are less common in diarrheagenic *E*. *coli* (< 3.2% of isolates identified). The spread of CTX-M-producing ETEC in the future may affect and hamper an antibiotic therapy of diarrhea caused by these pathogens [31].

Genetic determinants conferring resistance to antibacterials of some other drug classes (aminoglycosides and sulfonamides) were revealed into the class 1 integron In54 carrying the *dfrA17-aadA5* (GenBank KM236803) genetic cassette and class 1 integron In27 carrying the *dfrA12-orfF-aadA2* (GenBank KM236804) genetic cassette in two isolates ETEC_85-17V and ETEC_Ef-6 ccorrespondingly, and the class 1 integron carrying unidentified genetic cassette in ETEC_Ef-7 isolate (Table 2). According to reported data, integron structures are widespread in ETEC genomes, class 1 and class 2 integrons have been detected in 35% and 18% of the strains, respectively [32]. Of all our LT-producing *E*. *coli* strains, there was a single strain ETEC_Ef-6 that can be considered as a multidrug-resistant (MDR) bacterial pathogen carrying concurrently beta-lactam resistance genes and resistance cassettes to some other classes of antibacterials (aminoglycosides, sulfonamides). Interestingly, the other five ETEC isolates collected from the same patient Ef varied notably by the phenotype of their antimicrobial susceptibility and genetic markers (Table 2). The obtained results are consistent with earlier publications that there is no direct link between a sequence-type of ETEC and a serogroup they belong to, and there is no connection between the presence of pathogenic factors (enterotoxins and adhesion factors) and resistance genes in bacterial genomes [16].

## Conclusion

In this study we characterized the molecular biological properties of LT-producing ETEC strains which caused food-borne diseases in two large geographically remote cities of Russia. Collected strains were attributed to eleven novel sequence-types and to ST4 (ST10 Complex) which commonly spread among ETEC bacteria. The fact that ETEC strains belonging to ST4 and O6 serogroup were isolated from a patient and from sour cream allowed one to consider sour cream as a potential source of the food-borne outbreak. A correlation between ETEC sequence-types and the serogroups they belong to, as well as between the presence of pathogenicity genes and antimicrobial resistance was not found. The emergence of multi-drug resistant ETEC is of special concern and may pose serious problems to public health within the next few years. In conclusion, it should be emphasized that genetic heterogeneity of ETEC strains identified in the study can be explained by a high degree of *E*. *coli* genetic plasticity and the possibility of horizontal transfer of virulence factors and antibacterial resistance genes in populations of bacteria colonizing humans.

## Supporting Information

S1 Fig LT-detection using the latex agglutination assay. (PDF)
