# Supplementary material for: Molecular Characterization of Enterotoxin-Producing Escherichia coli Collected in 2011–2012, Russia
Source: PLoS One. 2015 Apr 29;10(4):e0123357. doi: 10.1371/journal.pone.0123357 (PMC4414545; doi:10.1371/journal.pone.0123357)

S1 Fig. LT-detection using the latex agglutination assay: 1 – positive control, purified cholera toxin, 2 g/L (Oxoid, UK); 2 - negative control, bacterial filtrate of the non-LT-producing *E. coli* strain HB101; 3 – bacterial filtrate of the ETEC_27-1; 4 - ETEC_73-7; 5 - ETEC_Ef-4; 6 - ETEC_Ef-6 strains.

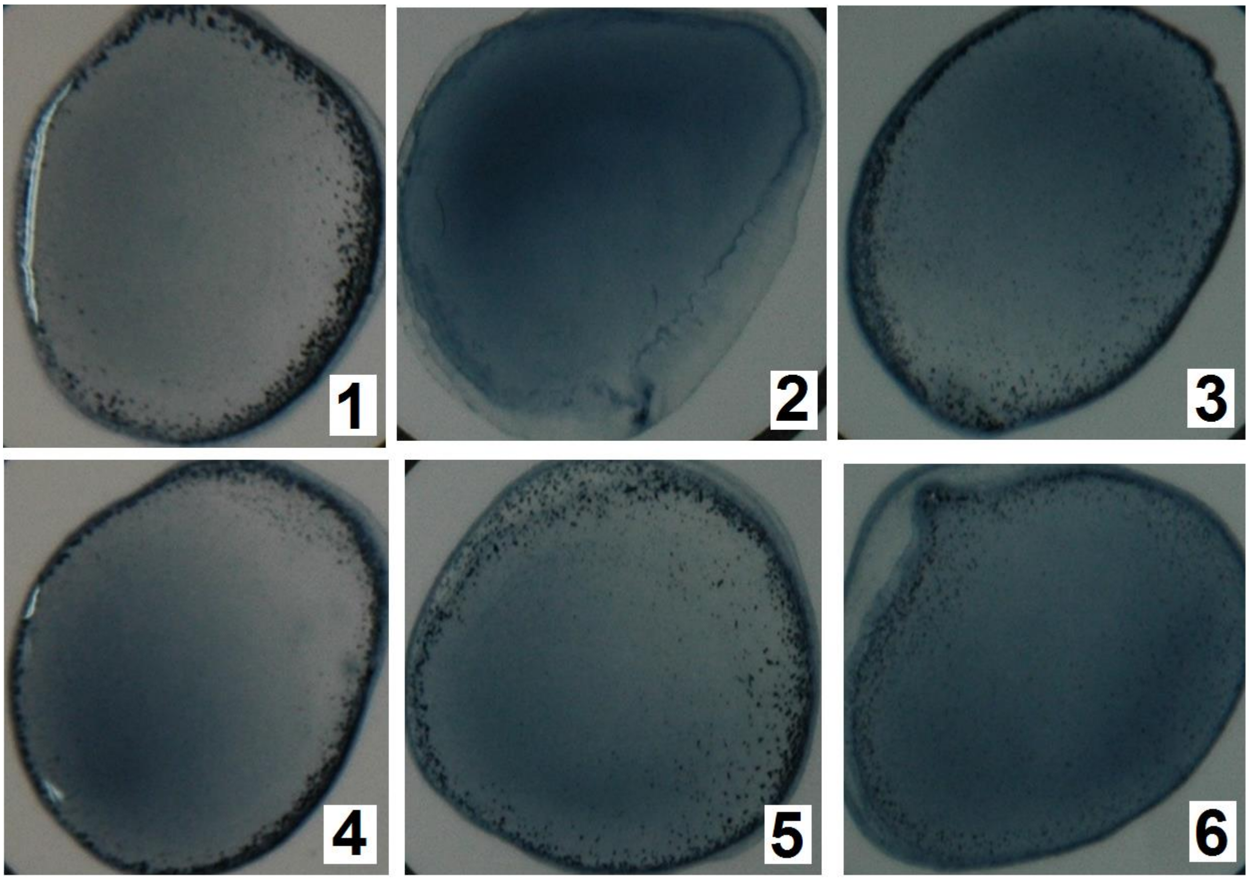

Supplement: S1 Fig — (PDF) [file pone.0123357.s001.pdf]
